# Early-life activities mediate the association between family socioeconomic status in early childhood and physical fitness in early adolescence

**DOI:** 10.1038/s41598-021-03883-8

**Published:** 2022-01-07

**Authors:** Rosa S. Wong, Keith T. S. Tung, Bianca N. K. Chan, Frederick K. W. Ho, Nirmala Rao, Ko Ling Chan, Jin Sun, Hung Kwan So, Wilfred H. S. Wong, Winnie W. Y. Tso, Jason C. S. Yam, Ian C. K. Wong, Patrick Ip

**Affiliations:** 1 Department of Paediatrics and Adolescent Medicine, The University of Hong Kong, Pokfulam Road, Hong Kong, China; 2 Institute of Health and Wellbeing, University of Glasgow, 1 Lilybank Gardens, Glasgow, G12 8RZ UK; 3 Faculty of Education, The University of Hong Kong, Hong Kong, SAR China; 4 Department of Applied Social Sciences, The Hong Kong Polytechnic University, Hong Kong, China; 5 Department of Early Childhood Education, The Education University of Hong Kong, Hong Kong, SAR China; 6 Department of Ophthalmology and Visual Sciences, The Chinese University of Hong Kong, Hong Kong, SAR China; 7 Department of Pharmacology and Pharmacy, Centre for Safe Medication Practice and Research, The University of Hong Kong, Hong Kong, SAR China; 8 Research Department of Practice and Policy, UCL School of Pharmacy, London, UK

**Keywords:** Risk factors, Psychology and behaviour, Socioeconomic scenarios, Paediatrics, Public health

## Abstract

The graded association between family socioeconomic status (SES) and physical fitness is evident, but little is known about the mechanism underlying this association. This study investigated the role of early-life activities as mediators of the longitudinal relationship between early-life SES and health-related physical fitness in 168 adolescents (51.2% boys; final mean age: 12.4 years old). In Wave 1 (2011–12), their parents completed questionnaires about family socioeconomic status (SES), parent–child activities, and child screen time. In Wave 2 (2014–15), participants’ physical activity levels were assessed through parent proxy-reports. In Wave 3 (2018–19), a direct assessment of handgrip strength, standing long-jump, and 6-min walk test (6MWT) performance was conducted. After controlling for demographic factors, results of mediation analyses revealed that (a) Wave 1 SES predicted Wave 3 long-jump and 6MWT performance; (b) child physical activity level in Wave 2 mediated the relation between Wave 1 SES and standing long-jump performance in Wave 3; and (c) recreational parent–child activities and child screen time in wave 1 mediated the relation between Wave 1 SES and 6MWT performance in Wave 3. Our findings suggest that the type and frequency of early-life activities play a role in the graded association between childhood SES and physical fitness in adolescence.

## Introduction

Family socioeconomic status (SES) is a composite measure of parental education, occupation and income, all of which are known to impact child development^1^. It has been reported that children from low-SES families were more difficult to reach their full potential due to the lack of nurturing care and exposure to developmentally appropriate activities, which was associated with poorer motor development^2^. Motor development is often assessed by levels of physical fitness^3^, which can be subdivided into health-related physical fitness and performance-related fitness. Physical fitness includes cardiorespiratory endurance, muscle strength, body composition and flexibility, whereas performance-related fitness reflects one’s balance, coordination, speed and agility^4^. For example, handgrip strength is a good indicator of the maximal isometric hand strength and upper limb muscle strength^5,6^; standing long-jump performance is reflective of lower limb explosive strength capacity^7^; and 6-min walk test (6MWT) performance is a reliable and valid measure of muscle tolerance and endurance^8,9^. It has been reported that children and adolescents from high-SES families were more likely to have higher mean fitness and less likely to have low/poor fitness^10^.

## Physical activity as a potential mediator of the association between SES and physical fitness

Previous longitudinal research has demonstrated a positive reciprocal relationship between physical activity and physical fitness across childhood through early adolescence^11^. A systematic review and meta-analysis of 23 studies also demonstrated a positive association between vigorous-intensity physical activity and cardiorespiratory fitness among children and adolescents^12^. Given the health benefits of physical activities, guidelines from the World Health Organization recommend children and adolescents to engage in at least 60 min of moderate- to vigorous-intensity physical activity each day^13^. However, individuals from low-SES backgrounds were found to have a higher likelihood of being physically inactive^14^. Children from lower-SES families may also have fewer access to outdoor play equipment such as bicycles and jump ropes and green areas^15,16^. The lack of these outdoor play opportunities in turn could have negative effects on motor development^17,18^.

## Recreational activities as a potential mediator of the association between SES and physical fitness

Although the link between physical activity and physical fitness is evident, much less is known about the potential effect of other recreational activities on physical fitness. Notably, in early childhood, physical activities are usually unstructured and performed through engagement in other types of recreational activities such as singing and storytelling. A previous study showed that participation in a developmentally appropriate music and movement program was associated with improvement in jumping and dynamic balance among preschoolers^19^. On the other hand, while drawing activities may not have direct effects on gross motor skill development, previous studies found that drawing skills were associated with executive functions such as inhibition and fine motor control^20^. These cognitive processes are also involved in exercise training and may relate to exercise motivation through self-regulation behavior such as the ability to start and stick to a workout routine amid other distractions^21^. For example, electronic devices are increasingly ubiquitous in today’s society and frequently used for online learning and entertainment purposes such that time for movement and play could be limited even in the preschool years^22^. Previous studies have demonstrated that frequent use of electronic devices is associated with a reduction in parent–child interaction time^23^. While health authorities recommend children and adolescents to limit their screen time to two hours or less per day^24^, evidence suggests that many Chinese children cannot meet this recommendation^25,26^. The risk of overuse of electronic devices is higher among children from disadvantaged families^27^. Electronic devices are also found more often in the bedroom of children from low-SES families^28^. Given that physical fitness involves multiple components, it is unclear whether different types of early-life activities would have similar or differential effects on these physical fitness components.

## The present study

Although there is evidence documenting the association between family SES and physical fitness, its underlying mechanism remains elusive with limited evidence on the predictive value of early-life activities for physical fitness in adolescence. It seems plausible that the impact of early-life SES on motor development may vary by the frequency and type of childhood activities. Hence, this study aimed to fill in this knowledge gap by examining the role of childhood activities in explaining the relationship between early-life SES and adolescent physical fitness.

## Results

### Descriptive statistics

Descriptive statistics for the study sample are presented in Table 1. They were 12.39 years old on average with 51.2% as boys and 48.8% as girls. A total of 65 (38.7%) had doctor-diagnosed physical diseases. In Wave 1, the average monthly household income of the study sample was USD 6282; 95% of the fathers and 60% of the mothers reported having a job. Regarding parental education level, 78% of the fathers and 79% of the mothers completed secondary education or above. In Wave 3, the mean (and SD) of handgrip strength was 35.59 ± 9.22 kg, whereas the mean (and SD) of 6MWT and standing long-jump performance were 600.58 ± 83.05 m and 130.29 ± 22.63 cm, respectively.

**Table 1:** Subject characteristics (n = 168).

Study variables	Participants assessed
Age at wave 3 health assessment (mean, SD)	12.39 (0.35)
Age at wave 1 assessment (mean, SD)	5.51 (0.30)
**Gender, n (%)**	
Boys	86 (51.2)
Girls	82 (48.8)
Having doctor-diagnosed physical diseases, n (%)	65 (38.7)
**Physical fitness (Wave 3)**	
Six-minute walk performance in meter (mean, SD)	600.58 (83.05)
Standing long-jump performance in centimeter (mean, SD)	130.29 (22.63)
Handgrip strength performance in kilogram (mean, SD)	35.59 (9.22)
**Socioeconomic status (Wave 1)**	
Family socioeconomic status index (mean, SD)	0.23 (0.98)
Monthly household income in USD (mean, SD)	6282 (3836)
**Maternal education level, n (%)**	
Bachelor’s degree or above	63 (37.5)
Grade 10 to diploma	69 (41.1)
Grade ≤ 9	36 (21.4)
**Father education level, n (%)**	
Bachelor degree or above	72 (42.9)
Grade 10 to diploma	59 (35.1)
Grade ≤ 9	35 (20.8)
Missing	2 (1.2)
**Maternal occupation at Wave 1, n (%)**	
Professionals	60 (35.7)
Non-professionals	41 (24.4)
Unemployed	17 (10.1)
Homemaker	31 (18.5)
Missing	19 (11.3)
**Father occupation at Wave 1, n (%)**	
Professionals	114 (67.9)
Non-professionals	46 (27.4)
Unemployed	4 (2.4)
Homemaker	0 (0.0)
Missing	4 (2.4)

### Associations between Wave 1 family SES and Wave 3 fitness test performance

Results showed that higher Wave 1 family SES predicted better 6MWT performance in Wave 3 in both unadjusted (β(95%CI) = 13.62(0.74, 26.50), p = 0.038) and adjusted (β(95%CI) = 14.54(1.60, 27.48), p = 0.028) models. After adjusting for child age, gender, and history of physical disease, higher Wave 1 family SES predicted better standing long-jump performance in Wave 3 (β(95%CI) = 3.97(0.39, 7.56), p = 0.030). The association between Wave 1 family SES and Wave 3 handgrip strength performance was not significant before and after adjusting for the demographic confounders.

### Bivariate correlations

In addition to the significant correlations with Wave 1 family SES (Table 2), when examining associations between early-life activities and fitness levels, better 6MWT performance was associated with higher CPCIS—recreational activities score (r = 0.19, p < 0.05) and less screen time (r = -0.20, p < 0.05) in Wave 1. Better standing long-jump performance correlated with higher PARCY level in Wave 2 (r = 0.35, p < 0.001), whereas handgrip strength performance did not significantly correlate with any of the early-life activities.

**Table 2:** Correlation matrix for study variables.

		1	2	3	4	5	6
1	Family socioeconomic status (Wave 1)	–	–	–	–	–	–
2	Recreational parent–child activities (Wave 1)	0.22**	–	–	–	–	–
3	Screen time (Wave 1)	−0.19*	−0.13	–	–	–	–
4	Physical activity level (Wave 2)	0.26**	0.18*	−0.08	–	–	–
5	6-min walk test (Wave 3)	0.16*	0.19*	−0.20*	0.14	–	–
6	Long jump test (Wave 3)	0.14**	0.07	−0.03	0.35***	0.14	–
7	Handgrip strength test (Wave 3)	−0.09	0.00	0.006	0.05	0.06	0.26***

*p < 0.05; **p < 0.01; ***p < 0.001.

### Pathways from Wave 1 family SES to Wave 3 standing long-jump performance

**Figure 1:**
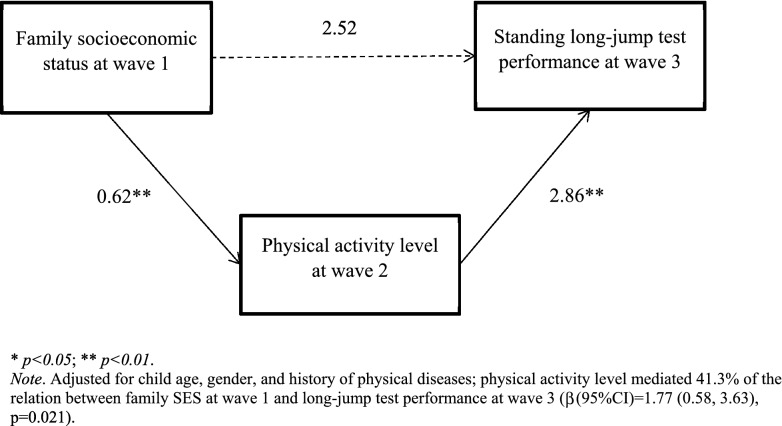
Path model linking family SES at Wave 1 to standing long-jump test performance at Wave 3.

After adjusting for confounders, higher Wave 1 family SES predicted better long-jump performance in Wave 3 (β(95%CI) = 4.29(0.68, 7.89), p = 0.020). Figure 1 illustrates the path model from Wave 1 family SES to Wave 3 long-jump performance, and Table 3 provides the effect estimates for each path. We found that after adjusting for Wave 2 PARCY level, the direct effect of Wave 1 family SES on Wave 3 long-jump performance was not significant (β(95%CI) = 2.52 (-1.17, 6.17), p = 0.181). On the other hand, the indirect effect of Wave 1 family SES on Wave 3 long-jump performance through Wave 2 PARCY level was significant (β(95%CI) = 1.77 (0.58, 3.63), p = 0.021). Specifically, higher Wave 1 family SES predicted an increase in Wave 2 physical activity level which in turn led to better Wave 3 long-jump performance.

**Table 3:** Path estimates for the model linking family SES at Wave 1 and standing long-jump test performance at Wave 3.

	β﻿ (95% CI)	p-value
Total effect (family SES—> long jump test)	4.29 (0.68,7.89)	0.020
Direct effect (family SES—> long jump test)	2.52 (−1.17, 6.17)	0.181
Indirect effect (family SES—> long jump test)	1.77 (0.58, 3.63)	0.021
Path (family SES—> physical activity level)	0.62 (0.22, 0.99)	0.002
Path (physical activity level—> long jump test)	2.85 (1.23, 4.46)	0.001

*SES* socioeconomic status; adjusted for child age, gender, and history of physical diseases.

### Pathways from Wave 1 family SES to Wave 3 6MWT performance

**Figure 2:**
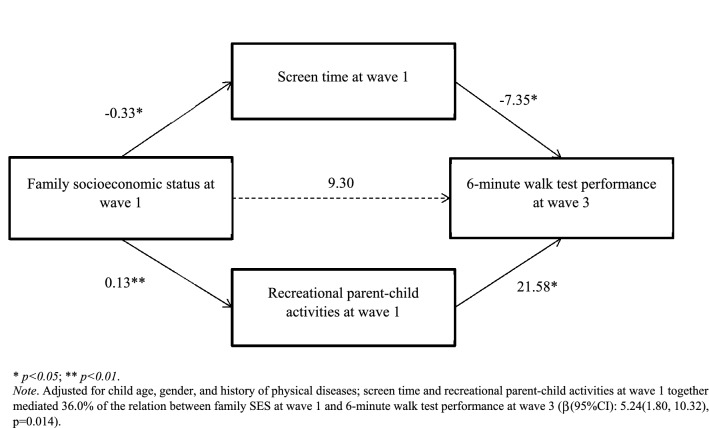
Path model linking family SES at Wave 1 to 6-min walk test performance at Wave 3.

After adjusting for confounders, higher Wave 1 family SES predicted better 6MWT performance in Wave 3 (β(95%CI) = 14.54(1.75, 27.63), p = 0.028). Figure 2 illustrates the path model from Wave 1 family SES to Wave 3 6MWT performance, and Table 4 provides the effect estimates for each path. The model involving two mediators showed significant indirect effects (β(95%CI): 5.24(1.80, 10.32), p = 0.014), but the direct effect of Wave 1 family SES on 6MWT performance in Wave 3 was not significant (β (95%CI) = 9.30(-3.55, 22.70), p = 0.170). In the fitness-enhancing pathway, higher family SES was associated with higher frequency of recreational parent–child activities in Wave 1 which in turn predicted better 6MWT performance in Wave 3. In the fitness-depressing pathway, lower family SES was associated with more screen time in Wave 1 which in turn predicted worse 6MWT performance in Wave 3.

**Table 4:** Path estimates for the model linking family SES at Wave 1 and 6-min walk test performance test performance at Wave 3.

	β (95% CI)	p-value
Total effect (family SES—> 6-min walk test)	14.54 (1.75, 27.63)	0.028
Direct effect (family SES—> 6-min walk test)	9.30 (−3.55, 22.70)	0.170
Indirect effect (family SES—> 6-min walk test)	5.24 (1.80, 10.32)	0.014
Path (family SES—> screen time)	−0.33 (−0.62, −0.05)	0.024
Path (family SES—> recreational parent–child activities)	0.13 (0.04, 0.23)	0.006
Path (screen time—> 6-min walk test)	−7.35 (−13.46, −1.13)	0.020
Path (recreational parent–child activities—> 6-min walk test)	21.58 (2.83, 39.83)	0.024

Adjusted for child age, gender, and history of physical diseases.

## Discussion

Using a prospective longitudinal design, this research is unique in examining the influence of early-life SES on health-related physical fitness and the role of childhood activities as potential mediators of this relationship. We found that a higher level of family SES in early childhood predicted a higher level of lower limb explosive strength (as reflected by better standing long-jump performance) and a higher level of lower limb muscle endurance (as reflected by better 6MWT performance) in early adolescence. While the effect of early-life SES on lower limb explosive strength was mediated by physical activity level in middle childhood, its effect on lower limb muscle endurance occurred through recreational parent–child activities and screen time in early childhood. By contrast, the effect of early-life SES on upper limb muscle strength (as reflected by handgrip strength performance) was small and not significant. Our results indicate that children from lower-SES families could have a higher likelihood of engaging in sedentary activities such as screen use over recreational and physical activities, which in turn can lead to poor physical fitness in adolescence.

While larger studies are needed to confirm the present results, the finding of early-life activities as mediators of the association between early-life SES and standing long-jump and 6MWT performance suggests that non-screen recreational activities are conducive to the training of lower limb explosive strength and muscle endurance. However, these recreational opportunities could be more limited among children from lower-SES families^15,16^. Effective home- and school-based lower limb muscle training programs would likely benefit underprivileged children. By contrast, the finding of no SES differences in handgrip strength performance suggests that improvement in upper limb muscle strength can be achieved irrespective of SES background. Future studies may consider exploring the effect of routine muscle-strengthening activities^29^ such as moving, lifting or carrying heavy things on upper limb muscle strength.

Previous research has reported that youth from higher-SES backgrounds were more likely to maintain regular sport participation^30^. Consistent with this finding, we observed higher physical activity level in middle childhood among those students from higher-SES families in early childhood. Also consistent with the previous finding of insufficient parental involvement in low-SES families^31^, adolescents from higher-SES families in this study were found to perform more recreational activities with parents in early childhood. Although this study did not measure the frequency of sport and physical activities in early childhood, the finding of a positive correlation between recreational parent–child activities in preschool years and physical activity level in primary school years adds to the literature that physical activity habits can be nurtured through engagement in general play-based parent–child activities at early ages. These activities can increase children’s awareness and understanding of the extent to which their parents are able and willing to support their hobbies and interests which is a key factor in promoting children’s physical activity level^32^.

In addition, it is generally agreed that activities in the early life stages have lasting effects on activity behaviors and developmental outcomes^33^. The associations of sedentary screen time with reduced muscle endurance and increased risk of health problems are evident^34^. Previous research also found an association between daily screen time at age 1 year and screen time later in life^35^. Our study provides additional evidence that early-life play-based activities can contribute to later gains in lower limb muscle endurance. This echoes the findings of a previous study reporting that participation in a range of developmentally appropriate, enjoyable and safe play-based activities through the early years is associated with better cardiorespiratory and musculoskeletal fitness and motor development^36^. It should be noted that many sports and fitness programs are effective in improving jumping performance and lower limb explosive strength^37^. We also found that physical activity level in middle childhood was predictive of lower limb explosive strength, but children from lower-SES families could have lower physical activity levels. School-based health education programs should be implemented to help children from disadvantaged backgrounds to gain access to physical activity^38^.

A major strength of our study was the use of longitudinal data across three time points from early childhood through adolescence which allowed for tracking the effect of early activities on long-term health-related outcomes. In addition, direct assessments were administered to measure three major health-related physical fitness components (upper limb muscle strength, lower limb muscle endurance, and lower limb explosive strength). This provides a comprehensive set of objective fitness data to increase the robustness of the present results. However, since the outcome, exposure, and mediator measures were collected in different waves, this study cannot infer causality, but temporality. Furthermore, we used parent-proxy reports to measure childhood activities, which could be subject to recall bias. Future research should use multiple-informants or direct observation methods to collect data on childhood activities. Another limitation is that we did not measure physical activity level in Wave 1, and thus we cannot draw any conclusion on the importance of recreational physical activity in early childhood as a potential predictor of physical fitness in adolescence. Furthermore, our study sample was not representative of Hong Kong population, as they came from only two local districts. Nonetheless, despite small sample size, significant relationships were still observed, which could give a helpful future direction for further research.

In conclusion, we found significant associations between family SES in early childhood and different components of health-related physical fitness in early adolescence. These associations were mediated by different types of childhood activities. Children from low-SES backgrounds, compared to the more affluent groups, could have weaker lower limb muscles due to insufficient activity levels. School-based programs incorporating lower limb muscle training should be encouraged. More studies are also needed to explore other mechanisms underlying the socioeconomic gradient in physical fitness using robust strategies such as large samples, objective activity measures, and multiple informants.

## Methods

### Participants

**Figure 3:**
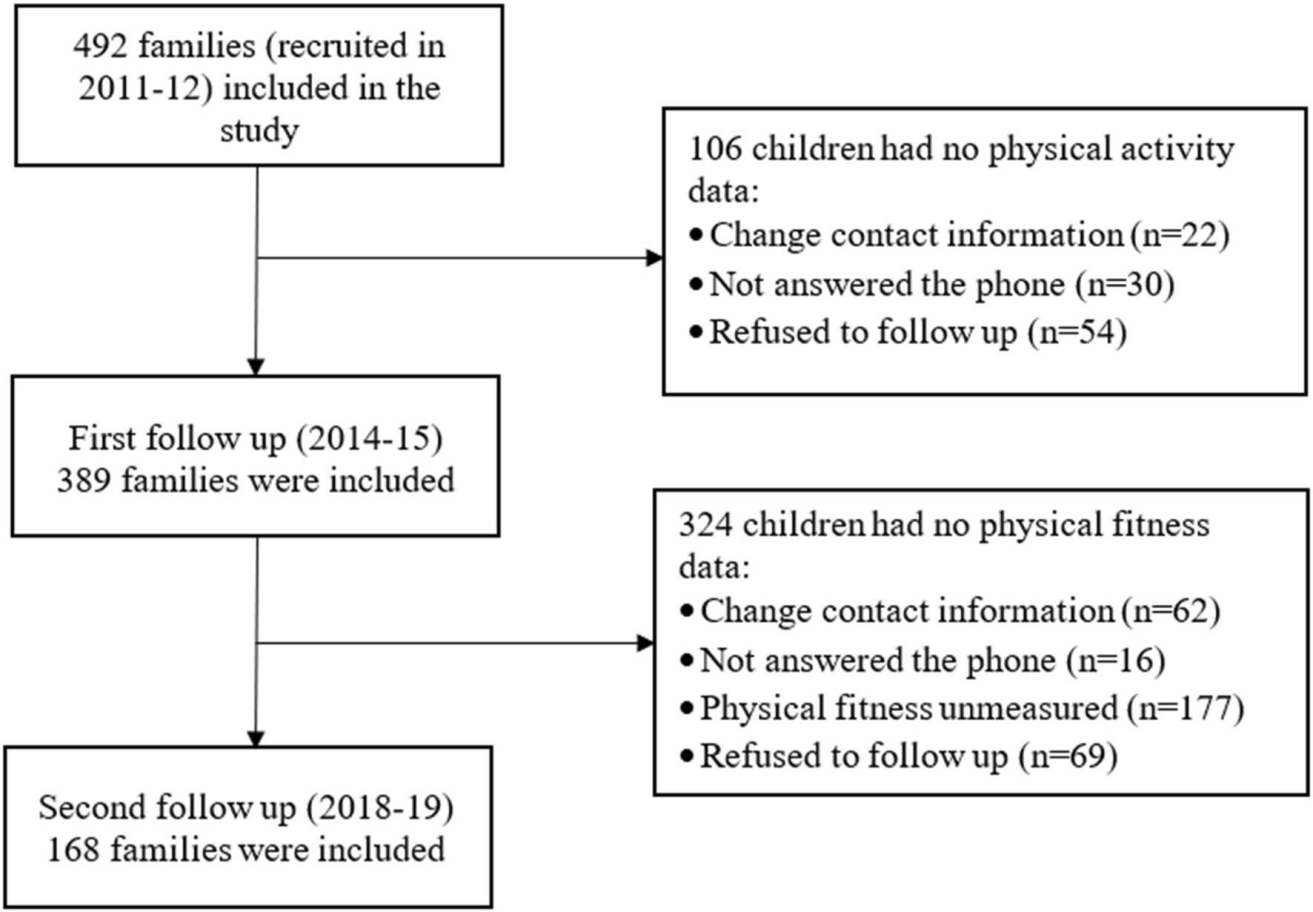
Study flow chart.

This study was part of a longitudinal cohort project aiming to examine the impact of early-life family SES on long-term health and development^39^. During the first year of project (2011), 22 kindergartens were randomly selected from two districts of Hong Kong (Hong Kong Island (an affluent district with about 41 kindergartens in total) and Yuen Long (a less wealthy district with about 71 kindergartens in total)), and 20 kindergartens (9 from Hong Kong Island and 11 from Yuen Long) agreed to participate. All students in the final year (K3) of these selected schools were eligible and invited to take part in the project. Three waves of data collection have been conducted. In Wave 1 when the child was in K3 (2011–12), 492 parents provided data on family demographics, screen time, and frequency of parent–child activities. In Wave 2 when the child was in Grade 3 (2014–15), 386 reported children’s physical activity level. In Wave 3 when the child was in Grade 7 (2018–19), 168 children performed the physical fitness tests. In Wave 1, the cohort was aged 5 to 6 years and 12 to 13 years in Wave 3. Only data of children who completed both Wave 1 and Wave 3 assessments were analyzed in this study. The flow chart is shown in Fig. 3. Written informed consents from parents/guardians were obtained prior to recruitment and data collection. Wave 3 completers and dropouts were similar in terms of children’s age and gender and parental education level and occupation and monthly household income in Wave 1.

### Exposure

#### Family socioeconomic status (Wave 1)

Family SES was analyzed as a family SES index z-score^39^. In Wave 1, information on family SES was collected via sociodemographic questionnaire and included items pertaining to four key SES factors: (1) maternal and paternal educational attainment; (2) maternal and paternal occupation; (3) family monthly income adjusted for household size; and (4) family asset score. A principal component analysis with varimax rotation was employed to combine these four SES-factors into a one-factor variable^40^. This SES index calculation method has been used in other local community and population studies^39,41,42^.

### Outcomes

#### Health-related physical fitness (Wave 3)

Three fitness tests were carried out during a health check-up: (i) handgrip test, (ii) 6MWT, and (iii) standing long-jump test. A trained research assistant first guided the adolescent participants how to perform each test, and the other research assistant recorded their performance. The participants performed each test separately from each other according to the given instructions as described below:

Upper limb muscle strength was measured in kilograms using a handgrip dynamometer (TKK 5101 Grip D; Takey, Tokyo, Japan; precision 0.1 kg). Each student squeezed gradually and continuously for at least 2 s while standing with arms straight down the side and without the dynamometer touching any part of the body except the hand being measured. The test was performed on each hand twice in a random order. The best measurement for each hand was recorded and the sum of the best measurement scores achieved by both hands was used in the analysis^43^. This is a reliable and valid procedure for both healthy and clinical populations with an intraclass correlation coefficient ranging from 0.92 to 0.96^44^.

Standing long-jump test with two-foot taking-off and landing was carried out to measure the explosive strength of lower limb with an acceptable reliability (intra-difference close to 0)^45^. Participants were asked to stand with feet slightly apart behind a line marked on the floor and to jump forward as far as possible. Three attempts were allowed. The best distance between the take-off line and the point where the back of heel is landing on was used in the analysis^46^.

Lower limb muscle endurance was assessed by the 6MWT. Participants were asked to walk as far as possible along a 30-m office corridor for 6 min. The 6-min walk distance was measured once and recorded in meters^47^. This test is considered reliable and valid for assessing exercise tolerance and endurance with an intraclass correlation coefficient of 0.94^48^.

### Mediators

#### Recreational parent–child activities (Wave 1)

The Chinese Parent–Child Interaction Scale (CPCIS)—Recreational Activities subscale, a validated parent–child interaction measure in Hong Kong with good internal consistency (Cronbach’s alpha = 0.71)^49^, was completed by the parent in Wave 1 to assess the weekly frequency of the following parent–child activities: reading, drawing, singing, storytelling, and discussing news and current affairs^39^. Items were rated on a 4-point scale ranging from 0 = none to 3 = 4 times or above per week and averaged to generate an overall mean score (M = 1.82, SD = 0.62), with higher scores representing more frequent recreational parent–child activities.

#### Screen time (Wave 1)

Children’s screen time was reported by a parent in Wave 1, with a screen time questionnaire designed to assess time spent watching television, video gaming on handheld and/or other types of game consoles, and using computers, tablet computers and smartphones for studying, gaming and/or web browsing. The child’s average daily screen time was calculated by averaging the amount of time reported for weekends and weekdays using the weighted average formula ([2 × weekend + 5 × weekday]/7) and reported in hours (M = 2.25, SD = 1.84). This calculation method has been used in previous community studies^50,51^.

#### Physical activity level (Wave 2)

In Wave 2, a parent completed the Physical Activity Rating Questionnaire for Children and Youth (PARCY) to report the child’s physical activity level on a 11-point scale ranging from 0 = no exercise at all in the last year to 10 = doing vigorous exercise almost every day in the last year (M = 5.34, SD = 2.19). The questionnaire consists of one assessment item that takes into consideration the physical activity frequency, duration, and intensity in the past year. It has been used in previous community and clinical epidemiological studies, showing good content validity index (90%) and test–retest reliability (0.86)^41,52^.

### Confounders

Children’s gender and date of birth were collected using the parent-completed sociodemographic questionnaire in Wave 1. During the health check-up in Wave 3, parents reported whether their children had any physician-diagnosed physical diseases. Children’s age at assessment was computed by subtracting date of birth from date of assessment. Informed by previous research^53^, age, gender, and history of physical diseases were associated with family SES and fitness levels and thus included as potential confounders in the mediation models.

### Data analysis

Descriptive statistics were first generated in SPSS version 25 (IBM corp, Armonk, NY, USA) to describe sample characteristics. In our analytic sample, missing data ranged from 1.19 to 11.31%. Assuming data were missing at random, we imputed missing data (5 imputed datasets) using multiple imputation by chained equations (MICE) R package^54^. All variables selected for analysis models were used to predict missing data. In addition to checking for missing values, continuous data were also checked for outliers and deviations from normality. Linear regression analyses were then performed to examine the unadjusted and adjusted relations between Wave 1 family SES and Wave 3 fitness test performance. Pearson’s correlations were also computed to assess the relationships between early-life family SES and activities and adolescent fitness outcomes. In order to test the hypothesis that the relations between early-life family SES and adolescent fitness outcomes were mediated by childhood activities, mediation models were performed, using Lavaan 0.6–5^55^ in R 3.6.3, on the basis of the regression and correlation results. Mediators in the model were Wave 1 screen time, Wave 1 CPCIS—recreational activities and Wave 2 PARCY level. All paths were adjusted for the potential influence of covariates in the model. In addition, we controlled for Wave 1 screen time, Wave 1 CPCIS—recreational activities or Wave 2 PARCY level depending on the model tested. The indirect effect is estimated by the product of the effect of Wave 1 family SES on the mediator and the effect of the mediator on the fitness outcome, given the direct effect of Wave 1 family SES and other covariates (Figs. 1 and 3). The indirect and direct effects and their 95% CI were estimated with 5000 bootstrap samples using the bias-corrected percentile method in Lavaan^55^. All the regression estimates (β) were unstandardized, with a p-value < 0.05 denoting statistical significance.

### Sample size estimation

Power analysis was conducted assuming two potential mediators. A path model with two potential mediators and small- to medium-sized path estimates (r = 0.25) would require a sample size of 146 to detect the two indirect effects at 0.05 significance level and 80% statistical power^56^.

### Ethical approval

The study protocol was approved by the Institutional Review Board of the University of Hong Kong/Hospital Authority Hong Kong West Cluster (UW 18-057). All research activities were performed in accordance with the Declaration of Helsinki. Informed consent was obtained from the parents or legal guardians of all participants.

## Abbreviations

SES: Socioeconomic status
6MWT: 6-Minute walk test
CPCIS: Chinese Parent–Child Interaction Scale
PARCY: Physical Activity Rating Questionnaire for Children and Youth
